# Differences in Posture, Neck Angle, and Body Discomfort During Various Electronic Device Usage with Virtual Classroom

**DOI:** 10.3390/ijerph22091418

**Published:** 2025-09-11

**Authors:** Roongnapa Intaruk, Praphatson Sengsoon

**Affiliations:** 1 Department of Physical Therapy, School of Allied Health Sciences, Walailak University, Nakhon Si Thammarat 80160, Thailand; 2 Movement Sciences and Exercise Research Center, Walailak University (MoveSE-WU), Nakhon Si Thammarat 80160, Thailand

**Keywords:** smartphone, tablet, notebook, posture, neck angle

## Abstract

Background: Prolonged use of electronic devices in virtual classrooms can influence posture, neck angle, and body discomfort. Recent evidence suggests that not only “incorrect” postures but also sustained static positions, regardless of being ergonomically correct, contribute to musculoskeletal strain. However, limited studies have directly compared posture and discomfort across different types of devices in a virtual classroom setting. Objective: To evaluate differences in posture, neck angle, and body discomfort among female university students during the use of three electronic devices (smartphone, tablet, notebook) in a virtual classroom for 20 min. Methods: Twenty-four healthy female participants (aged 18–23 years) completed three randomized sessions using a smartphone, tablet, or notebook in a virtual classroom task. Posture was assessed using the Rapid Upper Limb Assessment (RULA), neck angle was measured via motion analysis, and body discomfort was rated with a standardized visual analog scale. Statistical analyses were performed using repeated-measures ANOVA with Bonferroni correction, with effect sizes reported. Results: Significant differences were observed in posture (RULA scores: smartphone 5.12 ± 1.26; tablet 4.62 ± 1.35; notebook 4.21 ± 1.32, *p* < 0.05), neck angle (smartphone 32.48 ± 11.81 and tablet 36.93 ± 7.97, *p* > 0.05; notebook 39.30 ± 7.82, *p* > 0.05), and body discomfort of all regions (VAS: smartphone 1.08 ± 1.69; tablet 1.06 ± 1.75; notebook 1.01 ± 1.66, *p* < 0.05). Although all devices induced discomfort after 20 min of sustained posture, the smartphone condition showed the greatest neck flexion and discomfort. Conclusion: This study demonstrates that sustained posture during virtual classroom activities leads to increased neck angle deviation and body discomfort, with device type influencing the magnitude of these effects. These findings highlight the importance of postural variability and active breaks, rather than relying solely on maintaining a “correct” posture, to reduce musculoskeletal strain in technology-based learning environments.

## 1. Introduction

Online learning has become the primary mode of education in many countries, including Thailand, due to the COVID-19 pandemic, which forced educational institutions to close temporarily [1]. While online learning offers flexibility and accessibility, it also raises concerns regarding students’ physical health, particularly musculoskeletal issues associated with prolonged device use and poor workstation setup. Many students attend online classes and complete assignments using portable digital devices such as smartphones, tablets, and notebooks, each of which presents different ergonomic challenges.

The posture commonly adopted during virtual learning—particularly when using handheld devices—involves excessive neck flexion, forward head posture, and unsupported upper limbs. These positions increase biomechanical loads on the cervical and thoracic spine, contributing to musculoskeletal discomfort [2]. Young et al. [3] demonstrated that tablet use results in significantly greater neck flexion compared to notebooks, highlighting the influence of device type on posture and neck angle. In addition, survey data show that university students spend more than 6 h daily engaged in virtual classrooms and self-study, often without sufficient ergonomic awareness [4].

Smartphones, although the most accessible, are particularly problematic due to their small screen size and handheld nature, which force users into sustained postures associated with neck pain and body discomfort [5,6]. Similarly, tablets and notebooks, despite offering larger displays, are also linked with awkward postures because of their fixed screen–keyboard configuration and limited adaptability [7]. Epidemiological studies confirm that prolonged use of computers or digital devices exceeding four hours per day significantly increases the risk of work-related musculoskeletal disorders (WMSDs), including neck pain, back pain, and discomfort in the upper extremities [8,9]. Recent systematic reviews further indicate that sustained neck flexion, regardless of whether the posture is “correct,” contributes to cervical strain and reduced postural variability, emphasizing the role of both posture and duration of use in discomfort development [10].

Despite growing evidence, most prior studies have investigated single devices (e.g., smartphones or tablets) in isolation and under laboratory conditions, limiting their generalizability to actual virtual classroom settings. Few studies have directly compared posture, neck angle, and body discomfort across different devices during virtual learning among university students. This gap is important, since device type and usage context may differentially affect postural stress and discomfort.

Therefore, this study aims to evaluate and compare posture, neck angle, and body discomfort associated with the use of smartphones, tablets, and notebooks in a virtual classroom setting among female university students. The findings may inform ergonomic recommendations and preventive strategies to reduce WMSDs in technology-based learning environments.

## 2. Materials and Methods

This quasi-experimental study was designed to compare differences in posture, neck angle, and body discomfort while using different electronic devices in a virtual classroom setting. The study was conducted in accordance with the Declaration of Helsinki and approved by the Institutional Review Board (Ethics Committee) of Walailak University (WUEC-22-187-01, 20 June 2022). All participants provided written informed consent prior to participation.

A total of twenty-four female undergraduate students aged 18–23 years, with a body mass index (BMI) between 18.5 and 22.9 kg/m^2^, voluntarily participated in the study. All participants were right-hand dominant and had at least one year of experience using smartphones, tablets, and notebooks, as well as prior experience in virtual learning for at least one year. Female participants were selected to maintain a homogeneous study population and to reduce variability related to sex- and age-related differences in musculoskeletal characteristics, posture, and perceived discomfort.

Inclusion criteria were enrollment as a female undergraduate student aged 18–23 years, BMI 18.5–22.9 kg/m^2^, right-hand dominant, at least one year of experience with smartphones, tablets, and notebooks, and at least one year of prior virtual learning experience.

Exclusion criteria included visual impairments uncorrected to normal vision (20/20), musculoskeletal disorders of the upper limbs (e.g., chronic neck and shoulder pain, cervical disk herniation, or carpal tunnel syndrome), or a history of major injuries such as fractures or spinal limitations that could influence posture [11]. Participants with neurological disorders (e.g., cerebral palsy, stroke, partial paralysis) were also excluded to ensure consistency in neuromuscular function. Additional exclusions were the use of pain medication or muscle relaxants within six hours prior to testing, menstruation during the study period (to reduce variability in discomfort perception), pregnancy, and alcohol consumption within 24 h prior to testing [12].

These criteria were applied to minimize confounding factors that could alter postural behavior, neck angle, or perceived discomfort, thereby improving the reliability and consistency of the findings.

Each participant was evaluated in three workstation conditions, corresponding to the type of electronic device:

- Smartphone (6.1-inch display);
- Tablet (10.2-inch display);
- Notebook (16.5-inch display).

For each condition, participants were instructed to attend a 20 min instructional video session. During this period, their posture, neck angle, and body discomfort were assessed using validated methods:

- Posture: Rapid Upper Limb Assessment (RULA);
- Neck angle: Kinovea motion analysis software (Version 2023.1.1);
- Body discomfort: Modified Nordic Musculoskeletal Questionnaire (NMQ).

All measurements were conducted under standardized laboratory conditions to ensure consistency of device placement, seating, and lighting across participants.

### 2.1. Procedure

The experimental setup was standardized to ensure uniform conditions across all workstation trials. Each participant was seated in an adjustable chair with feet flat on the floor, back fully supported, and arms resting naturally.

#### 2.1.1. Workstation Setup

Smartphone and tablet: Placed on a table at an inclined angle to allow natural viewing while minimizing excessive neck flexion.

Notebook: Positioned with the screen at eye level to reduce neck strain.

Posture markers: Adhesive markers were placed on the spinous process of the C7 vertebra, tragus of the ear, and outer canthus of the eye to enable standardized measurement of craniocervical and craniovertebral angles.

#### 2.1.2. Measurement Protocol

Posture assessment: Postural risks were evaluated using the Rapid Upper Limb Assessment (RULA), based on images extracted from video recordings at 0, 10, and 20 min. The RULA of posture during electronic device use demonstrated excellent reliability. The intra-rater reliability was 0.882, and the inter-rater reliability was 0.970.

Neck angle: The Kinovea motion analysis software was used to calculate craniocervical and craniovertebral angles from standardized anatomical landmarks. The craniocervical angle and craniovertebral angle demonstrated excellent reliability. The intra-rater reliability for the craniocervical angle and craniovertebral angle were 0.879 and 0.881, respectively, while the inter-rater reliability were 0.956 and 0.957, respectively.

Body discomfort: Participants rated discomfort using the Modified Nordic Musculoskeletal Questionnaire (NMQ) before and after each workstation session, covering nine body regions (neck, shoulders, arms, wrists, upper back, lower back, hips, knees, and ankles).

#### 2.1.3. Experimental Sequence

Randomization: A blocked randomization method was applied, generating six possible sequences of device use to reduce order effects.

Pre-test: Before each session, participants completed an English proficiency test (three equivalent sets, rotated across workstations) to assess baseline knowledge of the instructional video content.

Video session: Participants watched a 20 min instructional video, during which posture and neck angle were recorded. They were permitted to take notes on paper provided by the researcher.

Post-test: Immediately after each video session, participants completed the same proficiency test again to confirm knowledge retention.

Body discomfort reporting: Participants rated their discomfort using the NMQ immediately after the post-test.

Rest period: A 10 min break (or longer if discomfort persisted) was provided before proceeding to the next workstation. Participants were instructed to rest quietly and avoid movements that could influence upper body posture.

#### 2.1.4. Data Extraction and Analysis

Still images at 0, 10, and 20 min were extracted from video footage for RULA and Kinovea analysis. Posture scores, neck angles, and body discomfort ratings were averaged and compared across devices. All data were compiled and subjected to statistical analysis to examine differences among smartphone, tablet, and notebook conditions.

### 2.2. Statistical Analysis

All statistical analyses were conducted using SPSS version 27 (IBM Corp., Armonk, NY, USA), with a significance level set at p < 0.05.

1. Normality testing: Data distribution was assessed using the Shapiro–Wilk test to determine whether parametric or non-parametric methods were appropriate.
2. Posture (RULA score): Differences in posture across the three electronic devices (smartphone, tablet, notebook) and time intervals (0, 10, and 20 min) were analyzed using a One-way repeated measures ANOVA. When significant effects were observed, Bonferroni post hoc correction was applied to adjust for multiple comparisons.
3. Neck angle (craniocervical and craniovertebral angles): Similarly to posture, neck angle variations were examined using a One-way repeated measures ANOVA with Bonferroni correction, comparing measurements at 0, 10, and 20 min across the three devices.
4. Body discomfort: Because discomfort ratings were not normally distributed, a Kruskal–Wallis test was employed to compare discomfort scores among the three devices. To assess within-device changes (pre-test vs. post-test), the Wilcoxon signed-rank test was used.

## 3. Results

A total of 24 female undergraduate students from Walailak University voluntarily participated in this study. Participants had a mean age of 21.42 ± 0.59 years, mean weight of 50.71 ± 4.42 kg, mean height of 159.63 ± 4.60 cm, and mean body mass index (BMI) of 19.95 ± 1.26 kg/m^2^. All participants were right-hand dominant and reported having more than one year of experience using smartphones, tablets, and notebooks in academic contexts.

Demographic characteristics of the participants are summarized in Table 1.

### 3.1. The Comparison of Postural Differences Using the Rapid Upper Limb Assessment (RULA) While Using Electronic Devices in a Virtual Classroom Was Conducted at 0, 10, and 20 min

**Figure 1:**
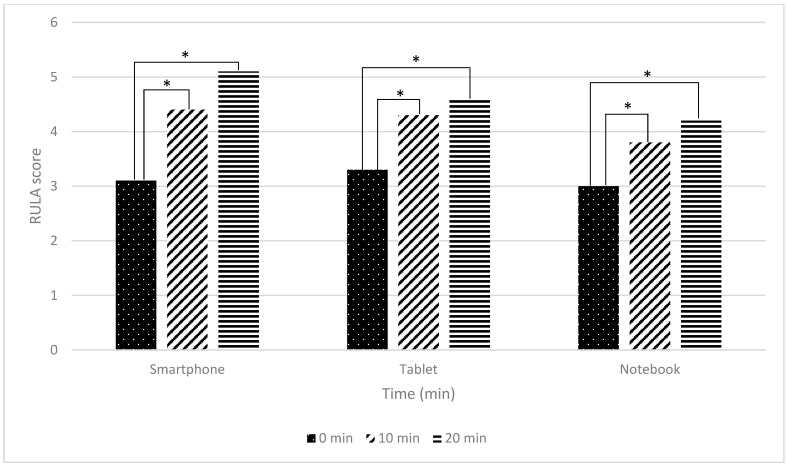
The graph illustrating the differences in posture comparison while using electronic devices in a virtual classroom at 0, 10, and 20 min (*n* = 24). Note: * indicates a statistically significant difference at *p* < 0.001, analyzed using One-way repeated measures ANOVA with Bonferroni correction.

The comparison of postural differences assessed by the Rapid Upper Limb Assessment (RULA) score across three electronic devices (smartphone, tablet, and notebook) are presented in Table 2 and Figure 1.

A between-group comparison of RULA scores among the three devices at 0, 10, and 20 min revealed no statistically significant differences (*p* > 0.05).

In contrast, the within-group analysis demonstrated significant postural deterioration over time (*p* < 0.001). Specifically, RULA scores increased significantly between 0 and 10 min and between 0 and 20 min of device use across all devices (*p* < 0.001), Bonferroni corrected, indicating progressive postural deterioration over time. Effect size analyses further supported these findings. For smartphones, a large effect was observed between 0 and 10 min (d = −0.920, *p* < 0.001), with an even larger effect between 0 and 20 min (d = −1.599, *p* < 0.001), suggesting substantial postural changes during prolonged use. Tablets showed a moderate-to-large effect between 0 and 10 min (d = −0.822, *p* < 0.001) and a large effect between 0 and 20 min (d = −1.047, *p* < 0.001), indicating a notable increase in ergonomic risk over time. For notebooks, large effects were also observed between 0 and 10 min (d = −0.896, *p* < 0.001) and 0 and 20 min (d = −0.941, *p* < 0.001), demonstrating similar patterns of posture deterioration.

However, no statistically significant differences were observed between 10 and 20 min (*p* > 0.05).

These results suggest that posture deteriorates markedly within the first 10 min of device use in a virtual classroom, regardless of device type, but tends to stabilize thereafter.

### 3.2. The Comparison of Neck Angle Differences While Using Electronic Devices in a Virtual Classroom Was Conducted at 0, 10, and 20 min

#### 3.2.1. The Comparison of Craniocervical Angle Differences

**Figure 2:**
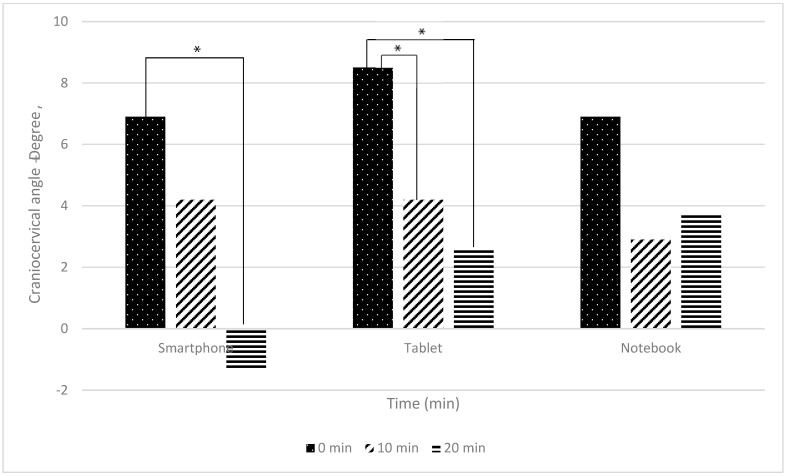
The graph illustrating the differences in craniocervical angle differences while using electronic devices in a virtual classroom at 0, 10, and 20 min (*n* = 24). Note: * indicates a statistically significant difference at *p* < 0.001, analyzed using One-way repeated measures ANOVA with Bonferroni correction.

The comparison of craniocervical angle differences across three electronic devices (smartphone, tablet, and notebook) are presented in Table 3 and Figure 2.

The between-group comparison of craniocervical angle across the three devices (smartphone, tablet, and notebook) at 0, 10, and 20 min revealed no statistically significant differences (*p* > 0.05).

For the within-group comparison, significant changes were observed for smartphone and tablet use (*p* < 0.05), but not for notebook use (*p* > 0.05). For smartphones, a significant reduction in craniocervical angle occurred between 0 and 20 min (d = 0.830, *p* < 0.05), indicating a notable forward shift in neck posture during prolonged use, whereas no significant changes were observed between 0 and 10 min or between 10 and 20 min (*p* > 0.05). Tablet use showed significant reductions in craniocervical angle between 0 and 10 min (d = 0.601, *p* < 0.05) and between 0 and 20 min (d = 0.690, *p* < 0.05), reflecting progressive neck flexion over time, while no differences were detected between 10 and 20 min (*p* > 0.05). In contrast, notebook use did not produce significant changes in craniocervical angle across any of the measured time intervals (*p* > 0.05), suggesting relative postural stability during use.

These results suggest that smartphone and tablet use are associated with progressive forward head posture during virtual classroom activities, while notebook use maintains a relatively stable craniocervical angle.

#### 3.2.2. The Comparison of Craniovertebral Angle Differences

**Figure 3:**
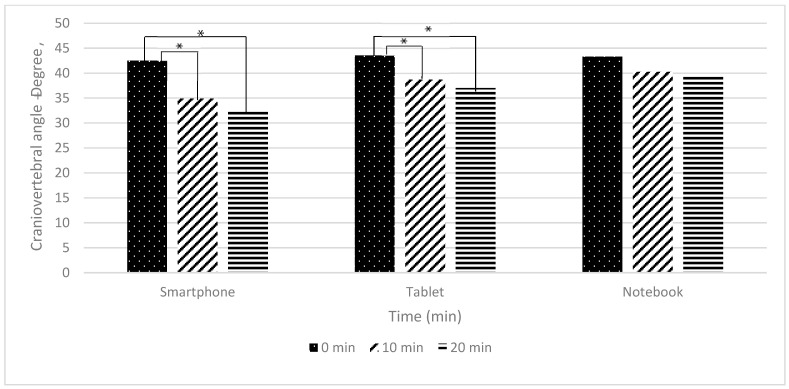
The graph illustrating the differences in craniovertebral angle differences while using electronic devices in a virtual classroom at 0, 10, and 20 min (*n* = 24). Note: * indicates a statistically significant difference at *p* < 0.001, analyzed using One-way repeated measures ANOVA with Bonferroni correction.

The comparison of craniovertebral angle differences across three electronic devices (smartphone, tablet, and notebook) are presented in Table 4 and Figure 3.

The between-group comparison of craniovertebral angle across the three devices (smartphone, tablet, and notebook) at 0, 10, and 20 min showed no statistically significant differences (*p* > 0.05).

For the within-group comparison, significant changes in craniovertebral angle were observed for smartphone and tablet use (*p* < 0.001), but not for notebook use (*p* > 0.05). For smartphones, significant reductions in craniovertebral angle occurred between 0 and 10 min, with a moderate-to-large effect (d = 0.710, *p* < 0.05), and between 0 and 20 min, with a large effect (d = 0.799, *p* < 0.05), indicating progressive forward head posture over time, whereas no significant change was observed between 10 and 20 min (*p* > 0.05). Tablet use exhibited a similar pattern, with significant reductions between 0 and 10 min (d = 0.750, *p* < 0.05) and between 0 and 20 min (d = 0.930, *p* < 0.05), while no significant differences were found between 10 and 20 min (*p* > 0.05). In contrast, notebook use did not produce significant changes in craniovertebral angle across any of the measured time points (*p* > 0.05), suggesting relative stability y of neck posture during use.

These findings indicate that smartphone and tablet use are associated with progressive forward head posture, while notebook use maintains a relatively stable craniovertebral angle during virtual classroom activities.

### 3.3. The Comparison of Body Discomfort Differences Before and After Using Electronic Devices in a Virtual Classroom Was Conducted to Evaluate Changes in Discomfort Levels

**Figure 4:**
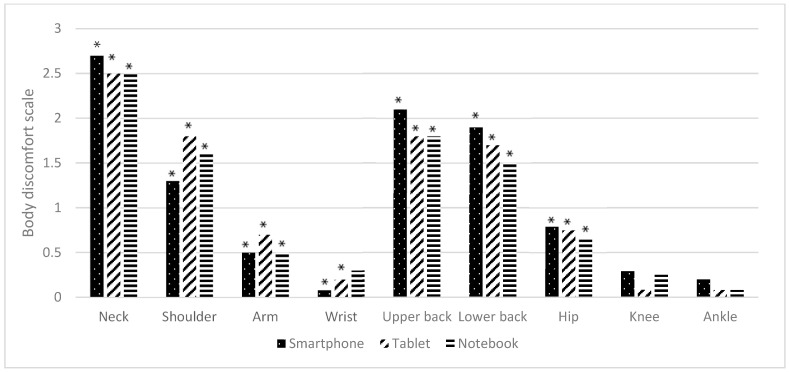
The graph illustrates the differences in body discomfort comparison before and after using electronic devices in a virtual classroom (*n* = 24). Note: * indicates a statistically significant difference at *p* < 0.05, analyzed using Wilcoxon signed-rank test.

The comparison of body discomfort differences before and after using electronic devices in a virtual classroom across three electronic devices (smartphone, tablet, and notebook) are presented in Table 5 and Figure 4.

The between-group comparison of body discomfort among smartphone, tablet, and notebook use showed no statistically significant differences (*p* > 0.05).

For the within-group comparison, significant increases in body discomfort were observed after device use for all three electronic devices (*p* < 0.05), although the affected body regions varied. Specifically:

Smartphones: Significant discomfort was reported in the neck, shoulders, arms, wrists, upper back, lower back, and hips, with moderate-to-large effects (d = 0.750–0.799, *p* < 0.05). No significant changes were observed in the knees and ankles (*p* > 0.05).

Tablets: Significant discomfort was reported in the neck, shoulders, arms, wrists, upper back, lower back, and hips, also with moderate-to-large effects (d = 0.750–0.799, *p* < 0.05). No significant changes were observed in the knees and ankles (*p* > 0.05).

Notebooks: Significant discomfort was reported in the neck, shoulders, arms, upper back, lower back, and hips, with moderate-to-large effects (d = 0.750–0.799, *p* < 0.05). The wrists, knees, and ankles did not show significant changes (*p* > 0.05).

These findings suggest that the pattern of musculoskeletal discomfort varies depending on the type of device used. Smartphones and tablets are associated with discomfort across multiple regions, particularly the wrists, while notebook use is associated with discomfort in fewer regions.

## 4. Discussion

This study compared posture, neck angles, and body discomfort while using smartphones, tablets, and notebooks in a virtual classroom among 24 right-handed female undergraduates. Participants were healthy and free from musculoskeletal disorders, ensuring that observed effects were primarily related to device use.

### 4.1. Postural Differences (RULA)

RULA scores at 0, 10, and 20 min showed no significant overall differences among devices (*p* > 0.05). However, smartphone use demonstrated a significant deterioration in posture over time (*p* < 0.05), with increased forward head posture and torso flexion, reflecting higher ergonomic risk. The small screen size and handheld position promoted sustained static postures and muscular strain, consistent with previous reports linking smartphone use to neck pain and musculoskeletal disorders [13,14,15]. Garud and Hande similarly highlighted that prolonged smartphone use elevates RULA scores and increases neck pain prevalence [16]. While all sustained device postures pose ergonomic risks, our findings indicate a tendency for posture-related strain to be more evident with smartphones than with tablets and notebooks, though these patterns should be interpreted cautiously given the lack of consistent between-device statistical differences.

### 4.2. Neck Angles

Craniocervical and craniovertebral angles showed no significant overall differences among devices at group level (*p* > 0.05). Nevertheless, prolonged smartphone use produced significant changes at 10 and 20 min (*p* < 0.05), indicating greater forward head flexion and increased neck strain [5,17,18]. Straker et al. reported similar findings, with screen height and workstation design significantly influencing neck posture, particularly in tablet users [19]. Tablets in this study also showed significant increases in neck flexion over time, in agreement with Young et al., who found that tablet inclination contributes to neck strain [3]. In contrast, notebooks showed minimal angular changes, likely due to larger screen size and better visual ergonomics [20]. However, extended notebook gaming has been associated with forward head posture risk [21]. Overall, while all devices carry ergonomic risks with sustained use, our results suggest a tendency for greater angular deviations during smartphone and tablet use, with comparatively smaller changes observed in notebooks. These patterns should be interpreted with caution given the lack of consistent between-device statistical differences.

### 4.3. Body Discomfort

No statistically significant overall differences in discomfort across nine body regions were observed between devices (*p* > 0.05), likely due to the controlled workstation setup [22]. Nonetheless, smartphone use was associated with significant increases in discomfort in the neck, upper back, and lower back (*p* < 0.05), consistent with Yang et al., who reported higher body discomfort with longer smartphone use [23]. Tablet use showed a similar pattern of increased discomfort, while notebooks were more often linked to discomfort in the neck, arms, and wrists [24,25]. These outcomes suggest that sustained use of all devices can provoke discomfort in different body regions, supporting prior evidence that prolonged visual display terminal (VDT) use and static postures contribute to musculoskeletal strain and risk of WMSDs [26].

## 5. Conclusions

Prolonged device use in virtual classrooms was associated with postural deterioration, increased neck angle deviations, and elevated body discomfort across all devices. Smartphones and tablets showed a tendency toward greater postural strain compared with notebooks, likely influenced by screen size and handheld or inclined positions. However, these patterns should be interpreted with caution, as between-device differences were not consistently significant. These findings highlight the importance of ergonomic education, appropriate workstation adjustments, and regular posture breaks to minimize musculoskeletal strain and reduce the risk of work-related musculoskeletal disorders among students engaged in prolonged online learning.

## 6. Clinical Application

This study demonstrates that prolonged use of electronic devices exerts measurable impacts on posture, neck angles, and body discomfort. While all devices carried ergonomic risks during sustained use, smartphones and tablets showed a tendency toward greater postural and musculoskeletal strain compared with notebooks, likely related to smaller screen sizes and handheld or inclined positions. These tendencies should be interpreted cautiously given the lack of consistent between-device statistical differences.

The findings provide practical implications for virtual learning and prolonged screen use. When smaller-screen devices must be used, ergonomic adjustments should be prioritized, such as positioning the device at eye level, aligning desk and chair height to maintain neutral spinal and lower extremity postures, and supporting the forearms during use. Additionally, incorporating regular breaks and posture changes can help mitigate muscle fatigue and discomfort, thereby reducing the risk of musculoskeletal strain.

## 7. Limitations

This study included only female undergraduate students, which may limit generalizability to males, whose anthropometric and musculoskeletal characteristics differ. The relatively short exposure duration (20 min) does not fully reflect real online learning sessions, which often extend for 60–120 min. Furthermore, standardizing device placement may have reduced ecological validity compared to everyday use, where users often do not optimize ergonomics. Finally, evaluator blinding was not feasible, which may have introduced bias. These limitations suggest that the observed tendencies across devices should be interpreted cautiously, and future research with more diverse populations, longer exposure times, and naturalistic setups is warranted.

## 8. Future Directions

Future research should examine longer exposure durations, mixed-gender and younger populations, and more naturalistic, uncontrolled device use. Investigating relationships between posture, neck angle, and upper-limb muscle activity with electromyography could provide deeper biomechanical insight. Longitudinal studies are also needed to clarify causal links between sustained electronic device use, postural adaptation, and musculoskeletal health outcomes, and to verify whether the tendencies observed across different devices hold true under real-world conditions.

## Figures and Tables

**Table 1:** General characteristics of participants (*n* = 24).

Variable	Mean ± SD	Minimum	Maximum
Age (year)	21.42 ± 0.59	21	23
Weight (kg)	50.71 ± 4.42	46	57
Height (cm)	159.63 ± 4.60	150	167
BMI (kg/m^2^)	19.95 ± 1.26	18.52	22.83

**Table 2:** The comparison of postural differences assessed by the Rapid Upper Limb Assessment (RULA) across three electronic devices.

Electronic Devices	0 min (Mean ± SD)	10 min (Mean ± SD)	20 min (Mean ± SD)	*p*
Smartphone	3.12 ± 0.45	4.38 ± 1.24	5.12 ± 1.26	<0.001 *
Tablet	3.29 ± 0.55	4.29 ± 1.33	4.62 ± 1.35	<0.000 *
Notebook	3.04 ± 0.69	3.83 ± 0.96	4.21 ± 1.32	<0.001 *

* indicates a statistically significant difference at *p* < 0.001, analyzed using One-way repeated measures ANOVA with Bonferroni correction.

**Table 3:** The comparison of craniocervical angle differences across three electronic devices.

Electronic Devices	0 min (Mean ± SD)	10 min (Mean ± SD)	20 min (Mean ± SD)	*p*
Smartphone	6.86 ± 5.18	4.23 ± 4.66	−1.33 ± 6.78	<0.001 *
Tablet	8.50 ± 5.34	4.25 ± 4.18	2.59 ± 5.31	<0.001 *
Notebook	6.86 ± 4.05	2.86 ± 4.02	3.68 ± 6.84	0.066

* indicates a statistically significant difference at *p* < 0.001, analyzed using One-way repeated measures ANOVA with Bonferroni correction.

**Table 4:** The comparison of craniovertebral angle differences across three electronic devices.

Electronic Devices	0 min (Mean ± SD)	10 min (Mean ± SD)	20 min (Mean ± SD)	*p*
Smartphone	42.50 ± 8.62	34.89 ± 10.41	32.48 ± 11.81	<0.001 *
Tablet	43.54 ± 7.46	38.68 ± 7.49	36.93 ± 7.97	<0.001 *
Notebook	43.29 ± 6.07	40.25 ± 5.41	39.30 ± 7.82	0.642

* indicates a statistically significant difference at *p* < 0.001, analyzed using One-way repeated measures ANOVA with Bonferroni correction.

**Table 5:** The comparison of body discomfort differences before and after using electronic devices in a virtual classroom across three electronic devices.

Electronic Devices	Body Discomfort Area								
	Neck	Shoulder	Arm	Wrist	Upper Back	Lower Back	Hip	Knee	Ankle
Smartphone	2.71 ± 2.31 *	1.29 ± 1.49 *	0.46 ± 0.83 *	0.08 ± 0.41 *	2.08 ± 2.17 *	1.88 ± 1.87 *	0.79 ± 1.28 *	0.29 ± 0.91	0.17 ± 0.48
Tablet	2.45 ± 2.45 *	1.75 ± 2.19 *	0.71 ± 1.52 *	0.21 ± 0.59 *	1.83 ± 1.87 *	1.71 ± 1.81 *	0.75 ± 1.39 *	0.08 ± 0.28	0.08 ± 0.41
Notebook	2.50 ± 2.48 *	1.62 ± 1.81 *	0.46 ± 0.88 *	0.25 ± 0.53	1.75 ± 1.85 *	1.50 ± 1.72 *	0.67 ± 1.43 *	0.25 ± 0.85	0.08 ± 0.41

* indicates a statistically significant difference at *p* < 0.001, analyzed using One-way repeated measures ANOVA with Bonferroni correction.

## Data Availability

The original contributions presented in this study are included in the article. Further inquiries can be directed to the corresponding author.
